# Airway Watch: A Rare Case of Adult Epiglottitis

**DOI:** 10.7759/cureus.73160

**Published:** 2024-11-06

**Authors:** Clates P Adams, Alec Ajhar, Dedra R Tolson

**Affiliations:** 1 Emergency Medicine, Madigan Army Medical Center, Tacoma, USA

**Keywords:** adult epiglottitis, epiglottitis, haemophilus influenzae, nasopharyngoscopy, vaccinations

## Abstract

Epiglottitis is an acute inflammatory condition involving the epiglottis and other supraglottic structures that may lead to airway obstruction. Historically, this condition primarily affected the pediatric population and was secondary to *Haemophilus influenzae* (Hib) infection. Since the vaccination program against Hib serotype B started at age two months, the number of affected pediatric patients has seen a drastic decline. Epiglottitis is now a condition that primarily affects adults. This case report presents a 58-year-old fully vaccinated female presenting with severe throat pain, odynophagia, subjective dyspnea, and chills for two days. During her evaluation, the patient underwent bedside nasopharyngoscopy by the emergency medicine team and was treated appropriately prior to evaluation by an otolaryngologist (ENT). The patient underwent a second nasopharyngoscopy with ENT 75 minutes after the original, which demonstrated vast improvements in the patient's clinical picture, allowing her to avoid intubation. This report highlights the importance of having epiglottitis on the differential for adults, the importance of the emergency medicine physician in performing nasopharyngoscopy, and early intervention with antibiotics and corticosteroids* to improve patient outcomes.

## Introduction

Epiglottitis is defined as an acute inflammatory condition involving the epiglottis and other supraglottic structures, often resulting from bacteriemia or invasion of the epithelial layer by a pathogenic agent that may lead to airway obstruction [1-3]. Epiglottitis is traditionally known as a condition primarily affecting the pediatric population, caused by *Haemophilus influenzae* (Hib). Since immunizations against Hib serotype B started at two months of age in 1987, the number of affected pediatric patients has rapidly declined [1,4,5].

Epiglottitis is now a condition that primarily affects adults aged 16-66, with ages 45-64 having the highest incidence rates, ranging from 0.6-3 cases per 100,000 adults and national mortality around 1% [1-3,6]. Adult epiglottitis is often caused by *Streptococcus pyogenes*, *Streptococcus pneumoniae*, *Staphylococcus aureus*, or *Pasteurella multocida* in immunocompetent adults, and *Pseudomonas aeruginosa* or Candida in immunocompromised adults [1,3,7]. Risk factors that enhance disease likelihood include hypertension, diabetes, and a history of smoking [1,2].

As children grow older, the size of the epiglottis decreases and becomes more rigid. For this reason, epiglottitis is less severe in adults. The disease process typically manifests in adulthood as supraglottitis, or inflammation of the supraglottic tissue, and is less likely to generate complications related to airway obstruction [3,4]. Patients classically present with pharyngitis, odynophagia, and voice changes [1,4]. Those who present with stridor, respiratory distress, dyspnea, tachycardia, tachypnea, or rapid onset (less than 24 hours) have a high likelihood of needing airway intervention [1,4].

## Case presentation

A 58-year-old Asian female, fully vaccinated, presented to the emergency department with two days of severe throat pain, odynophagia, subjective dyspnea, and chills. She had a prior medical history of breast cancer status post-chemotherapy, radiation, and radical mastectomy, as well as hypertension, hyperlipidemia, neuropathy, and a remote history of tobacco use. On day 1 of symptoms, the patient was able to tolerate oral intake but had developed difficulty tolerating her oral secretions and a muffled-sounding voice, as noted by her husband. Otherwise, the patient’s review of systems was negative, including recent travel or known sick contacts.

On physical examination, the patient was afebrile and hemodynamically stable (T: 37.0 °C (oral), heart rate: 92 beats per minute, respiration rate: 17, blood pressure: 140/96 mmHg, SpO_2_: 95% on room air). She appeared uncomfortable, with bilateral anterior cervical lymphadenopathy, post-nasal drip, an erythematous posterior oral pharynx, edematous tonsils without exudates, a muffled voice, pain with tracheal rocking, and anterior neck pain with full extension.

**Figure 1:**
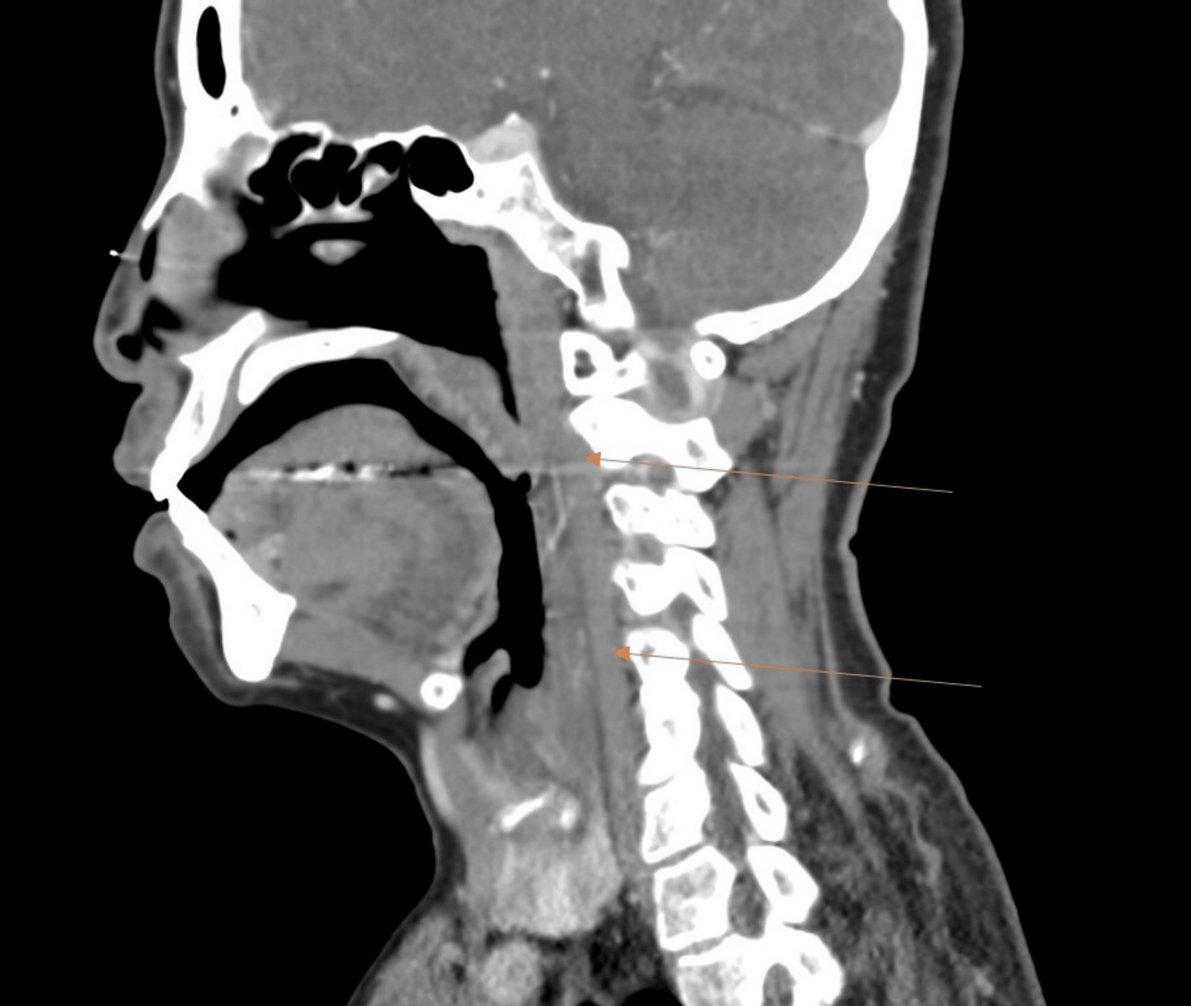
CT neck soft tissue with contrast: retropharyngeal edema from C2–C5. Superior arrow demonstrating start of edema. Inferior arrow demonstrating end of edema.

**Figure 2:**
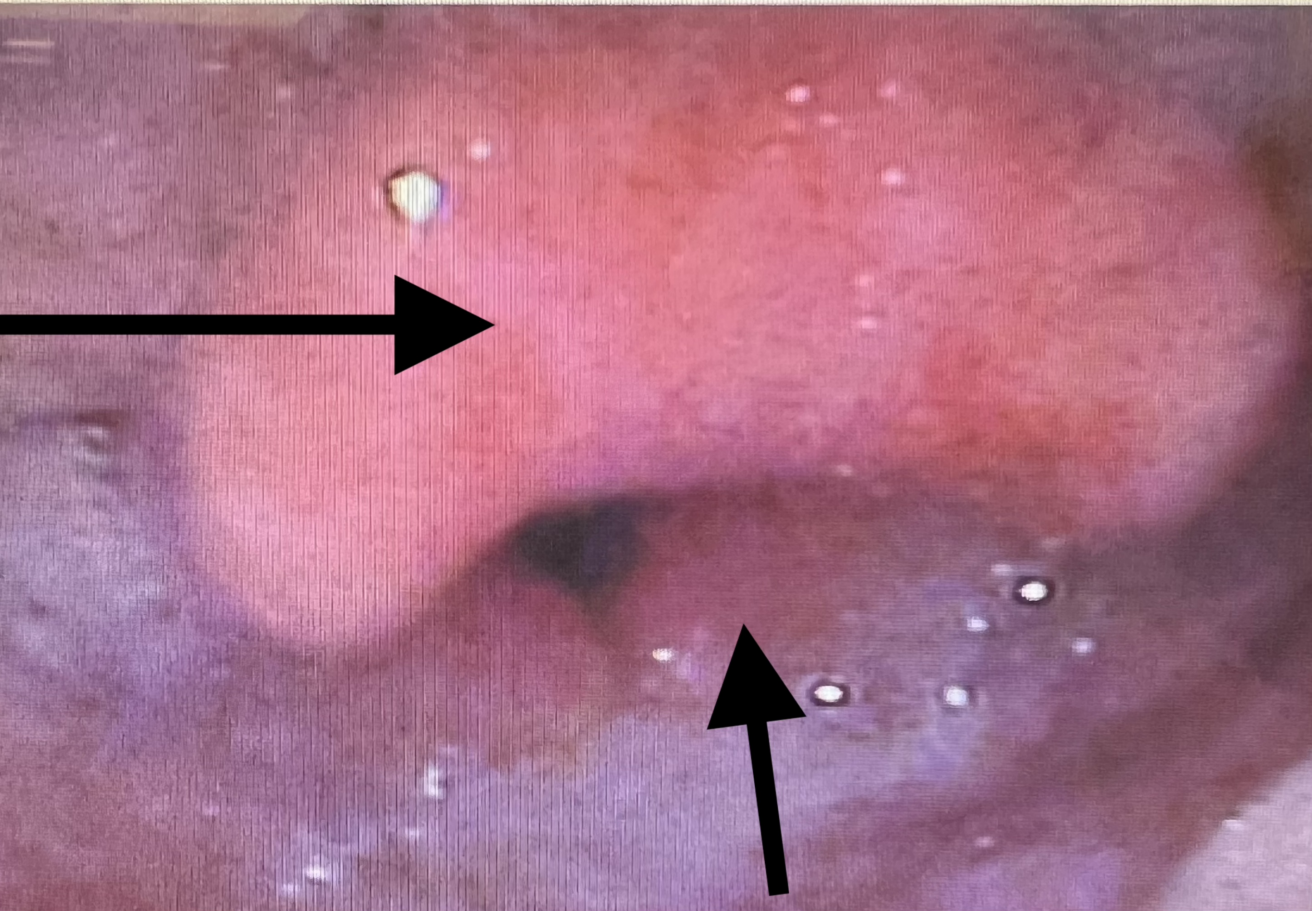
Nasopharyngoscopy performed by the ED team – evidence of inflammation of the epiglottis, supraglottic tissue, and arytenoids. Superior arrow - epiglottis, inferior arrow - supraglottic tissue.

**Figure 3:**
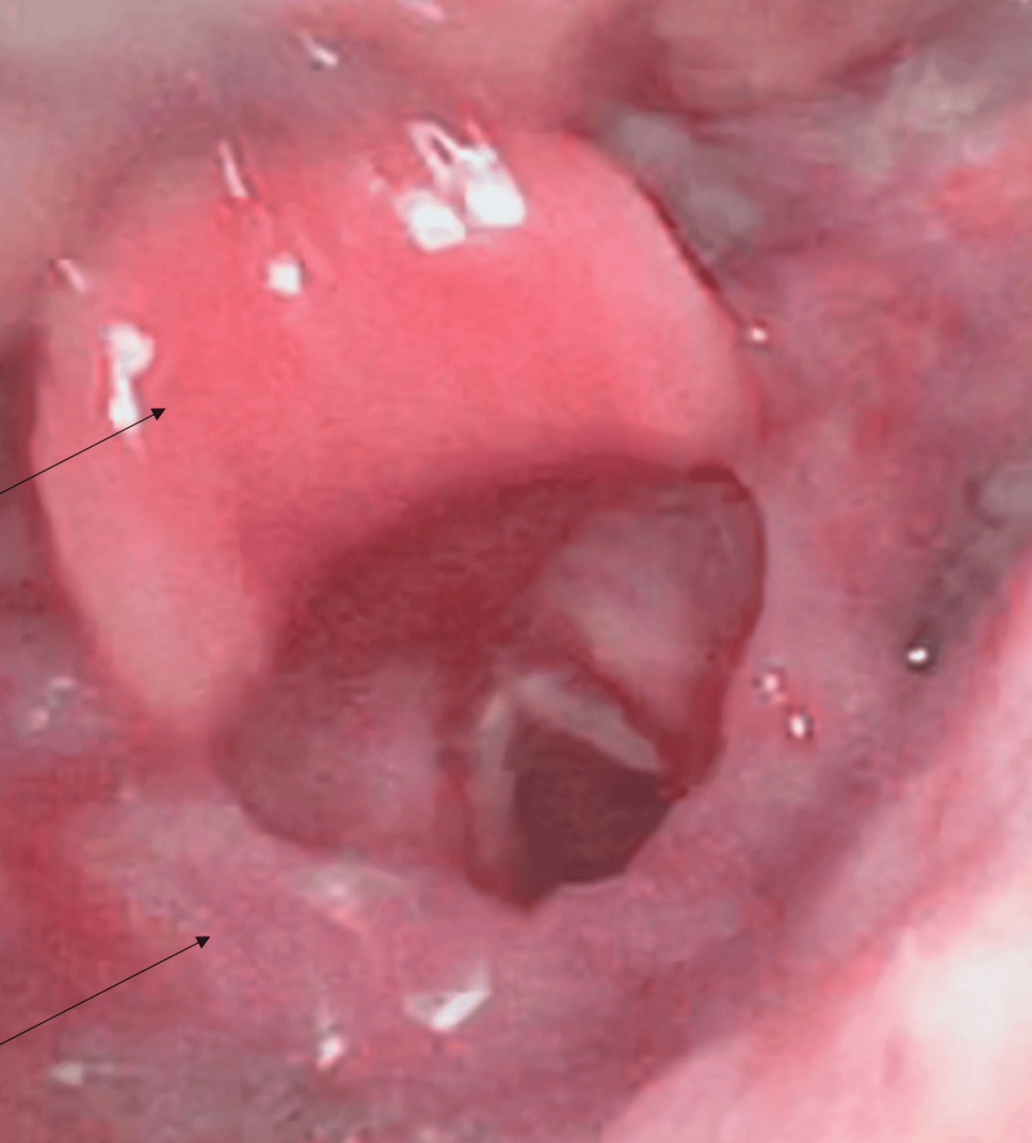
Nasopharyngoscopy by ENT 75 minutes after initial NP scope - reduction in inflammation of the epiglottis, supraglottic tissues, and arytenoids. Superior arrow - epiglottis, inferior arrow - supraglottic tissues.

A workup was initiated, which included a complete blood count (CBC), complete metabolic panel (CMP), erythrocyte sedimentation rate (ESR), C-reactive protein (CRP), procalcitonin, urinalysis (UA), blood cultures (bcx), point of care strep test (POC Strep), mononucleosis screen, extended respiratory film array, EKG, chest X-ray (CXR), and computed tomography (CT) neck soft tissue with IV contrast. Her CBC demonstrated leukocytosis, ESR was elevated, and CRP was elevated, but the remainder of her laboratory results were unremarkable, as can be seen in their respective attached tables, and she was not able to isolate a causative organism (Tables 1-4). CXR was negative for acute pathology. CT neck (Figure 1) demonstrated a small amount of retropharyngeal edema extending from C2 to C5. Given the nonspecific nature of the CT, the patient consented to a nasopharyngoscopy, which was then performed by the emergency medicine team. Prior to the examination, the patient was pretreated with intranasal Afrin and 4% intranasal lidocaine. The NP scope was introduced into the left nares, where the vocal cords and epiglottis were visualized (Figure 2), revealing an edematous epiglottis consistent with the diagnosis of acute epiglottitis. The patient was administered normal saline (1 L), dexamethasone (10 mg IV), acetaminophen (1 g IV), and a dose of ampicillin-sulbactam (3 g IV). Otolaryngology (ENT) was consulted, who performed a repeat nasopharyngeal exam of the patient 75 minutes later (Figure 3), which demonstrated moderate edema and erythema of the epiglottis and bilateral arytenoids suggestive of supraglottitis. ENT ultimately recommended intensive care unit (ICU) admission for airway watch, and the patient was admitted to the ICU with scheduled ampicillin-sulbactam (3 g every eight hours), dexamethasone (10 mg every eight hours for three doses), and daily labs to include CBC, ESR, and CRP.

**Table 1:** Complete blood count results.

CBC results	Patient value	Reference value
White blood cells (WBC)	14.2 × 10^3^/μL	4.5–13.0 × 10^3^/μL
Red blood cells (RBC)	4.70 × 10^6^ μL	3.8 0 5.10 × 10^6^ μL
Hemoglobin	14.5 g/dL	10.0–15.0 g/dL
Hematocrit	43%	34–45%
Mean corpuscular volume (MCV)	92 fL	80–98 fL
Mean corpuscular hemoglobin (MCH)	30.9 pg	26.7–33.7 pg
Mean corpuscular hemoglobin concentration (MCHC)	33.4 g/dL	32.5–37.5 g/dL
Red cell distribution width (RDW)	13.3%	11.5–15.0 %
Platelets	191 × 10^3^/μL	140–420 × 10^3^ μL
Mean platelet volume (MPV)	10.2 fL	7.0–12.0 fL
Neutrophil %	82.5 %	38.5–76.5%
Lymphocyte %	10.4%	14.0–46.0%
Monocyte %	6.0%	3.0–13.0%
Eosinophil %	0.7%	0.0–7.4%
Basophil %	0.1%	0.0–2.5%
Imm. granulocyte %	0.3%	0.0–2.0%
Neutrophil absolute	11.73 × 10^3^/μL	1.50–10.0 × 10^3^/μL
Lymph absolute	1.48 × 10^3^/μL	0.90–3.00 × 10^3^/μL
Mono absolute	0.85 × 10^3^/μL	0.20–0.90 × 10^3^/μL
Eosinophil Absolute	0.10 × 10^3^/μL	0.0–0.40 × 10^3^/μL
Basophil absolute	0.02 × 10^3^/μL	0.0–0.20 × 10^3^/μL
nRBC %	0.02%	0.0–5.0%
nRBC absolute	0.00 × 10^3^/μL	0.00–2.0 × 10^3^/μL
Imm. granulocyte absolute	0.04 × 10^3^/μL	0.0–2.0 × 10^3^/μL

**Table 2:** Complete metabolic panel results.

CMP results	Patient value	Reference value
Sodium	141 mmol/L	135–145 mmol/L
Potassium	3.60 mmol/L	3.50–5.10 mmol/L
Chloride	104 mmol/L	98–107 mmol/L
CO2	22 mmol/L	22–31 mmol/L
AGAP	15 mmol/L	7–16 mmol/L
Osmo Calc	294 mOsm/kg	277–308 mOsm/kg
BUN	17.0 mg/dL	6.0–23.0 mg/dL
Creatine level	0.85 mg/dL	0.50–1.0 mg/dL
BUN/creat ratio	20	7–25
eGFR	79 mL/min/1.73 m^2^	
Glucose	109 mg/dL	74–109 mg/dL
Calcium	8.7 mg/dL	8.6–10.3 mg/dL
Protein total	7.6 g/dL	6.6–8.7 g/dL
Albumin	4.4 g/dL	3.5–5.2 g/dL
A/G ratio	1.4 mg/dL	2.50–4.50 mg/dL
Bilirubin total	0.60 mg/dL	0.15–1.20 mg/dL
Bilirubin direct	0.2 mg/dL	0.0–0.3 mg/dL
Alk phos	79 U/L	35–104 U/L
ALT	27 U/L	0–33 U/L
AST	23 U/L	0–35 U/L

**Table 3:** Inflammatory markers.

Inflammatory markers	Patient value	Reference value
ESR	52 mm/hr	0–30 mm/hr
CRP	8.10 mg/dL	0.30–0.50 mg/dL
Procalcitonin	0.11 ng/mL	0.0–0.50 ng/mL
POC Strep	Negative	Positive or negative
Mononucleosis screen	Negative	Positive or negative
Blood cultures	Negative	Positive or negative

On day 2 of admission, the patient underwent a repeat nasopharyngeal exam performed by ENT, which demonstrated resolved edema of the epiglottis and improved edema of the bilateral arytenoids. Repeat CBC demonstrated a down-trending WBC (11.9), down-trending ESR, and down-trending CRP. The patient was discharged with seven days of amoxicillin-clavulanate.

**Table 4:** Extended respiratory film array results.

Extended respiratory film array		
Adenovirus	Not detected	Detected or not detected
Coronavirus HKU1	Not detected	Detected or not detected
Coronavirus NL63	Not detected	Detected or not detected
Coronavirus 229E	Not detected	Detected or not detected
Coronavirus OC43	Not detected	Detected or not detected
Human Rhinovirus/Enterovirus	Not detected	Detected or not detected
Influenza A	Not detected	Detected or not detected
Influenza B	Not detected	Detected or not detected
Parainfluenza 1	Not detected	Detected or not detected
Parainfluenza 2	Not detected	Detected or not detected
Parainfluenza 3	Not detected	Detected or not detected
Parainfluenza 4	Not detected	Detected or not detected
Respiratory syncytial virus	Not detected	Detected or not detected
Bordetella parapertussis	Not detected	Detected or not detected
Bordetella pertussis	Not detected	Detected or not detected
Chlamydia pneumoniae	Not detected	Detected or not detected
Mycoplasma pneumoniae	Not detected	Detected or not detected
Human metapneumovirus	Not detected	Detected or not detected
Resp PCR Interpretation	Not detected	Detected or not detected
SARS-CoV-2 PCR	Not detected	Detected or not detected

## Discussion

Through the widespread vaccination effort in pediatrics starting in 1987, epiglottitis is now a primary concern in the adult population, particularly those born before 1987. Additionally, with a recent movement of anti-vaccination, a high index of suspension for epiglottitis should remain on the differential of all emergency medicine physicians. Epiglottitis is a clinical diagnosis confirmed with direct visualization, which is preferred to be done in the operating room [1]. Neck radiographs demonstrating the classic “thumbprint” sign are poorly sensitive and should not be relied on for confirmation [1].

Treatment is focused on airway control, with 10.2% of patients requiring endotracheal intubation. In the cases necessitating intubation, awake fiberoptic intubation had the best success rate (100%), and awake laryngoscopy had the lowest success rate (52%) [6]. Prior to intubation, empiric treatment should be started and should include a third-generation cephalosporin, an anti-staphylococcal antimicrobial, glucocorticoid*, and a head of bed elevation greater than 30° [1,3]. Ceftriaxone (1 g IV daily) or ampicillin-sulbactam (3 g IV 4 times daily) are the recommended antibiotics, or if the patient is thought to have a severe infection or is immunocompromised, cefepime (2 g IV daily) plus vancomycin is recommended.

The use of glucocorticoids is controversial since no well-designed, double-blinded studies demonstrate benefits in morbidity or mortality [8]. Despite the controversy, most people would likely administer this class of medications, as there have been no clear negative outcomes.

## Conclusions

Epiglottitis is a condition that occurs primarily in adulthood. Acute treatment should remain focused on airway management, source control, antimicrobial therapy, and possibly corticosteroids. Finally, this case highlights the importance of nasopharyngoscopy for emergency medicine providers, allowing them to obtain and interpret real-time findings to potentially avoid an impending airway catastrophe.

## References

[1] Lindquist B, Zachariah S, Kulkarni A (2017). Adult epiglottitis: a case series. Perm J.

[2] Sajid A, Rafique MH, Khan F Case report: adult presentation of acute epiglottitis with severe airway complications - Emergency management and surgical intervention. Authorea.

[3] Yeruva K, Dave AD, Thota G, Mekala SR, Gudi TR (2023). A case of epiglottitis in an elderly man. Consultant.

[4] Ramlatchan SR, Kramer N, Ganti L (2018). Back to basics: a case of adult epiglottitis. Cureus.

[5] Shah RK, Stocks C (2010). Epiglottitis in the United States: national trends, variances, prognosis, and management. Laryngoscope.

[6] Booth AW, Pungsornruk K, Llewellyn S, Sturgess D, Vidhani K (2024). Airway management of adult epiglottitis: a systematic review and meta-analysis. BJA Open.

[7] Sofoulis L, Dawson B, Pham T (2024). Pasteurella multocida epiglottitis: a case report. JEM Reports.

[8] Phillips JS, Innes AJ, Naik MS (2004). Corticosteroids for supraglottitis. Br J Anaesth.

